# Supplementary material for: The NuRD component CHD3 promotes BMP signalling during cranial neural crest cell specification
Source: EMBO Rep. 2025 Aug 20;26(19):4723–41. doi: 10.1038/s44319-025-00555-w (PMC12508100; doi:10.1038/s44319-025-00555-w)
Supplement: Supplementary file 1 — Appendix [file 44319_2025_555_MOESM1_ESM.pdf]

Appendix - Table of contents

Table of contents……………..1
Appendix Figure S1………….2
Appendix Figure S2………….3
Appendix Figure S3………….4
Appendix Figure S4………….5
Appendix Figure S5………….6
Appendix Figure S6………….7
Appendix Figure S7………….8
Appendix Figure S8………….9
Appendix Figure S9………...10
Appendix Figure S10……….11
Appendix Figure S11……….12
Appendix Figure S12……….13

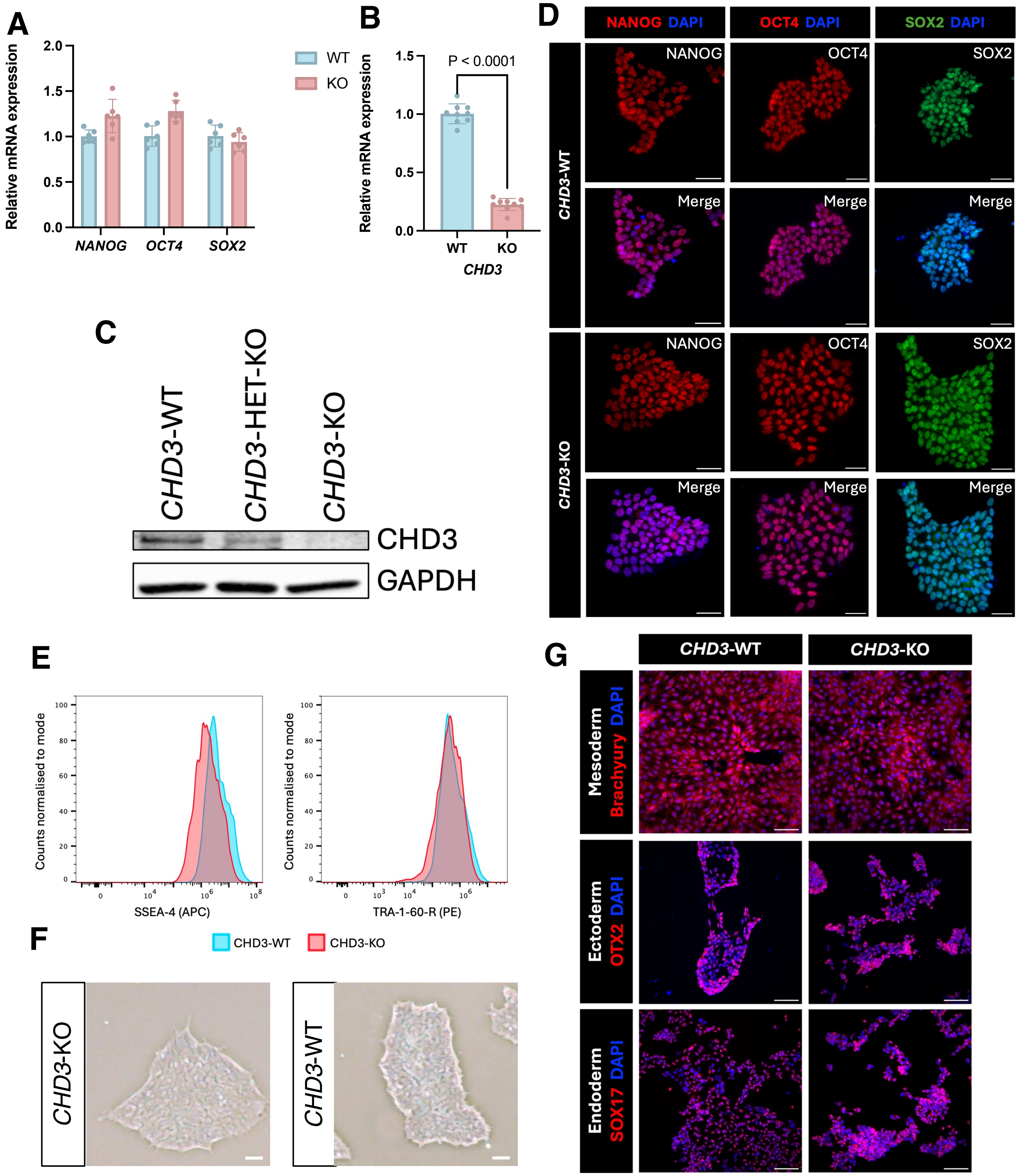

Appendix Figure S1. Validation of the iPSC lines. (A and B) RT-qPCR quantifying the relative expression levels of (A) pluripotency markers and (B) CHD3 in *CHD3*-WT (WT) and *CHD3*-KO (KO) iPSCs. Differences between lines were assessed using unpaired student's t-test with significant p-values displayed (n=6 with 3 technical replicates of 2 biological replicates for each sample). (C) Western blot for CHD3 in *CHD3*-WT, *CHD3*-HET-KO and *CHD3*-KO iPSCs. GAPDH is used as a loading control. (D) Immunofluorescence for key pluripotency markers in *CHD3*-WT and *CHD3*-KO iPSCs. Scale bar: 50 µm. (E) Flow cytometry for pluripotency surface markers in *CHD3*-WT and *CHD3*-KO iPSCs. (F) Example iPSC colonies from *CHD3*-KO and *CHD3*-WT lines. Scale bar: 50 µm.(G) Immunofluorescence for mesoderm marker brachyury, ectoderm marker OTX2 and endoderm marker SOX17 in *CHD3*-WT and *CHD3*-KO following differentiation into the three respective germ layers. Scale bar: 100 µm.

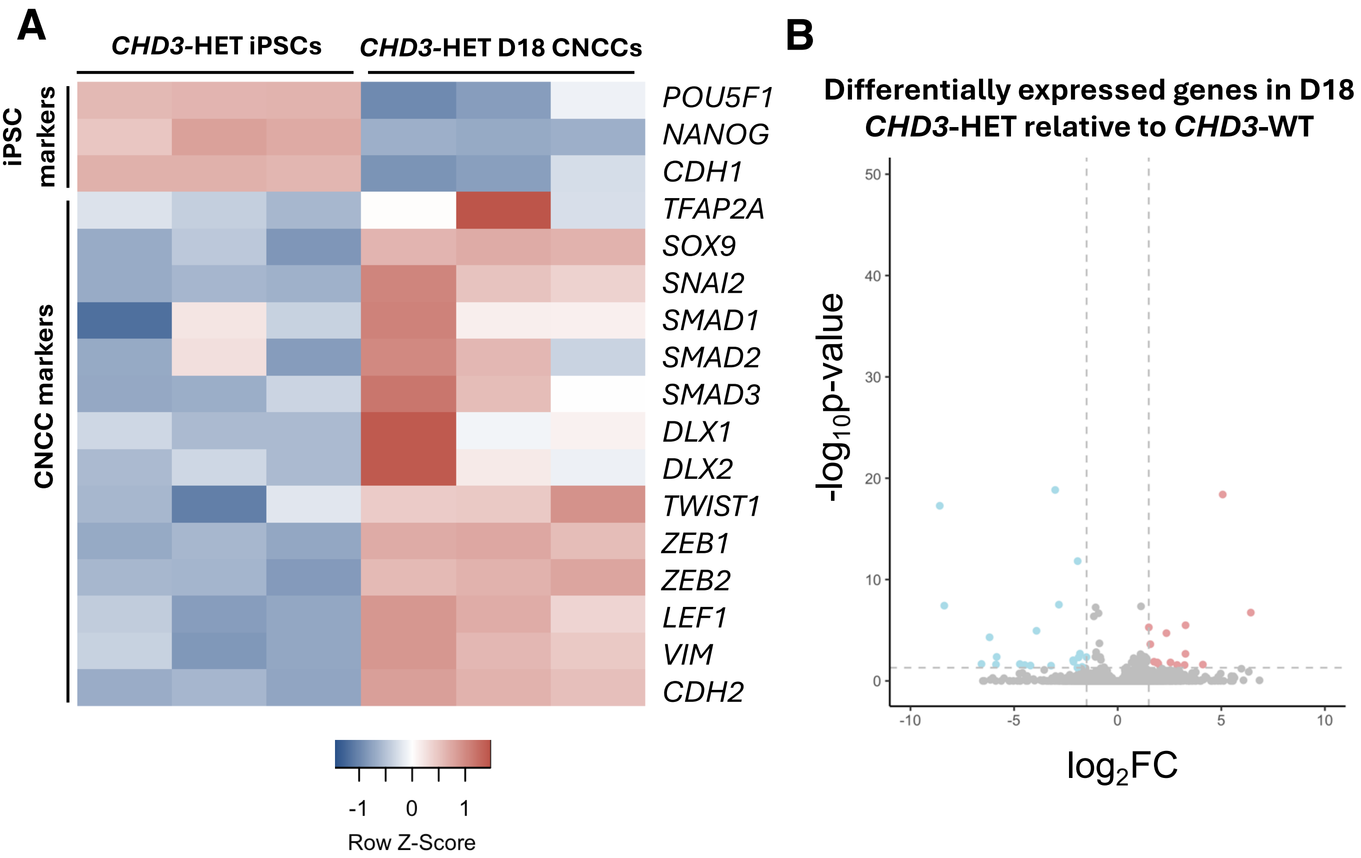

Appendix Figure S2. Characterisation of *CHD3*-HET-KO D18 CNCCs. (A) Heatmap displaying expression of key pluripotency markers and CNCC markers in *CHD3*-HET-KO iPSCs and *CHD3*-HET-KO D18 CNCCs. (B) Volcano plot of differentially expressed genes in *CHD3*-HET-KO comparative to *CHD3*-WT in D18 CNCCs. Blue dots represent downregulated genes with p-adj < 0.05 and log2FoldChange < -1.5. Red dots represent upregulated genes with p-adj < 0.05 and log2FoldChange > 1.5.

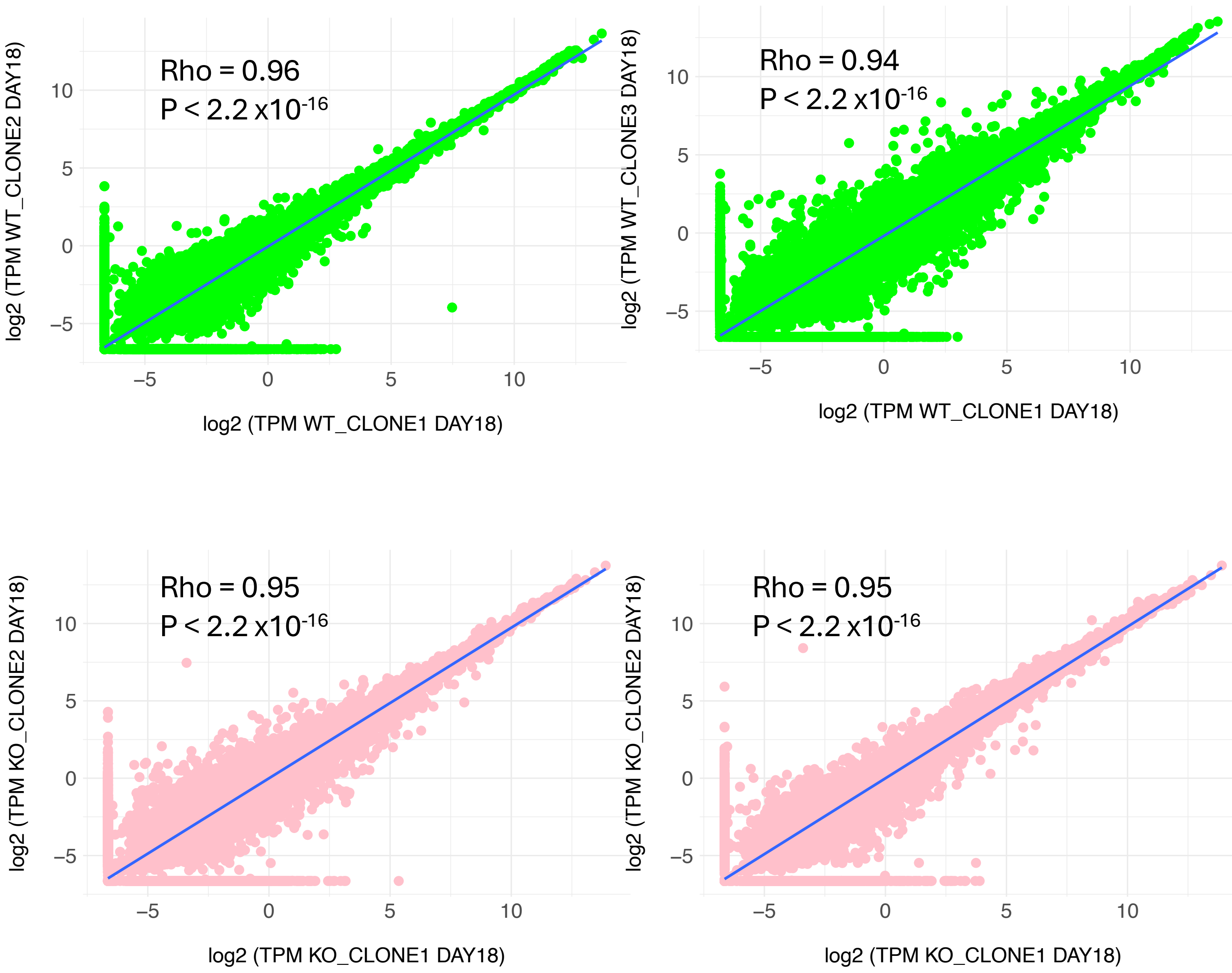

Appendix Figure S3. Correlation plots showing spearman correlation across RNA-seq biological replicates (i.e. *CHD3*-WT and *CHD3*-KO CRISPR-clones) at day 18 of iPSC-to-CNCC differentiation. Exact p-values determined from spearman's correlation test are displayed.

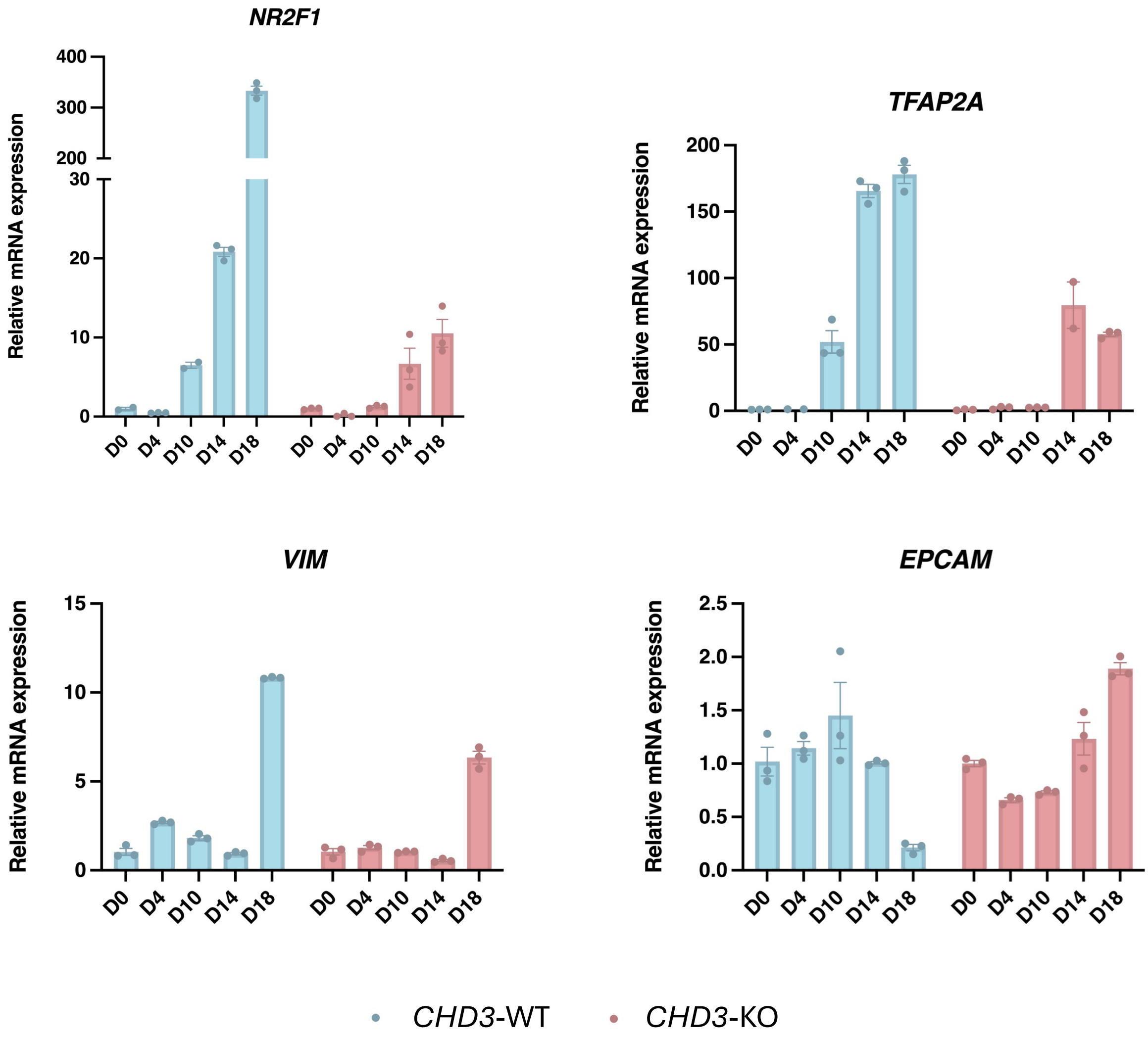

Appendix Figure S4. RT-qPCR assessing the relative expression levels of CNCC markers (*TFAP2A* and *NR2F1*), mesenchymal marker *(VIM)* and epithelial marker (*EPCAM*) between *CHD3*-WT and *CHD3*-KO cells at various timepoints during the differentiation of iPSCs to CNCCs. (n=3 technical replicates for each timepoint). Data are presented as mean ± SEM.

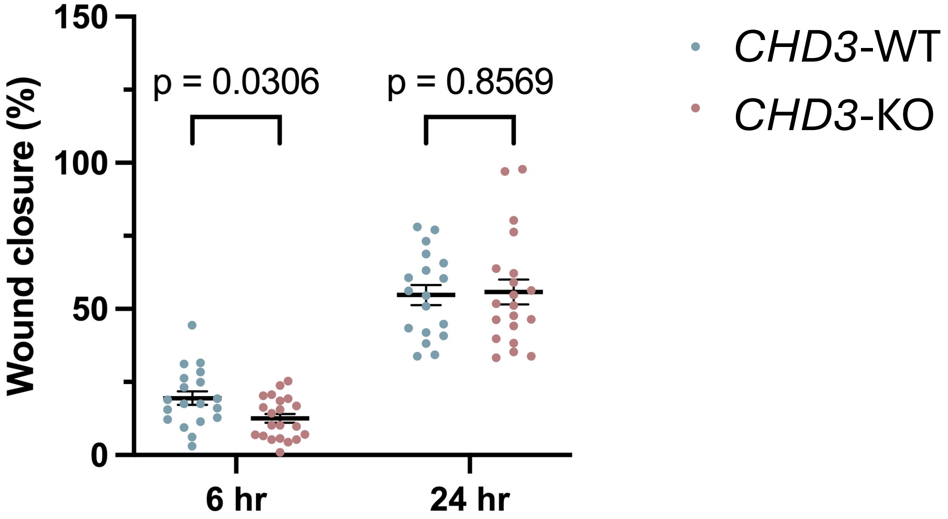

Appendix Figure S5. Scatter plot showing the percentage of wound closure 6 hours and 24 hours after the start of a scratch wound assay (n=24 independent replicates for each timepoint). *CHD3*-WT and *CHD3*-KO were compared at each time point using an unpaired student's t-test and exact p-values are displayed. Individual data points corresponding to one well in the scratch wound assay are plotted with lines indicating mean ± SEM.

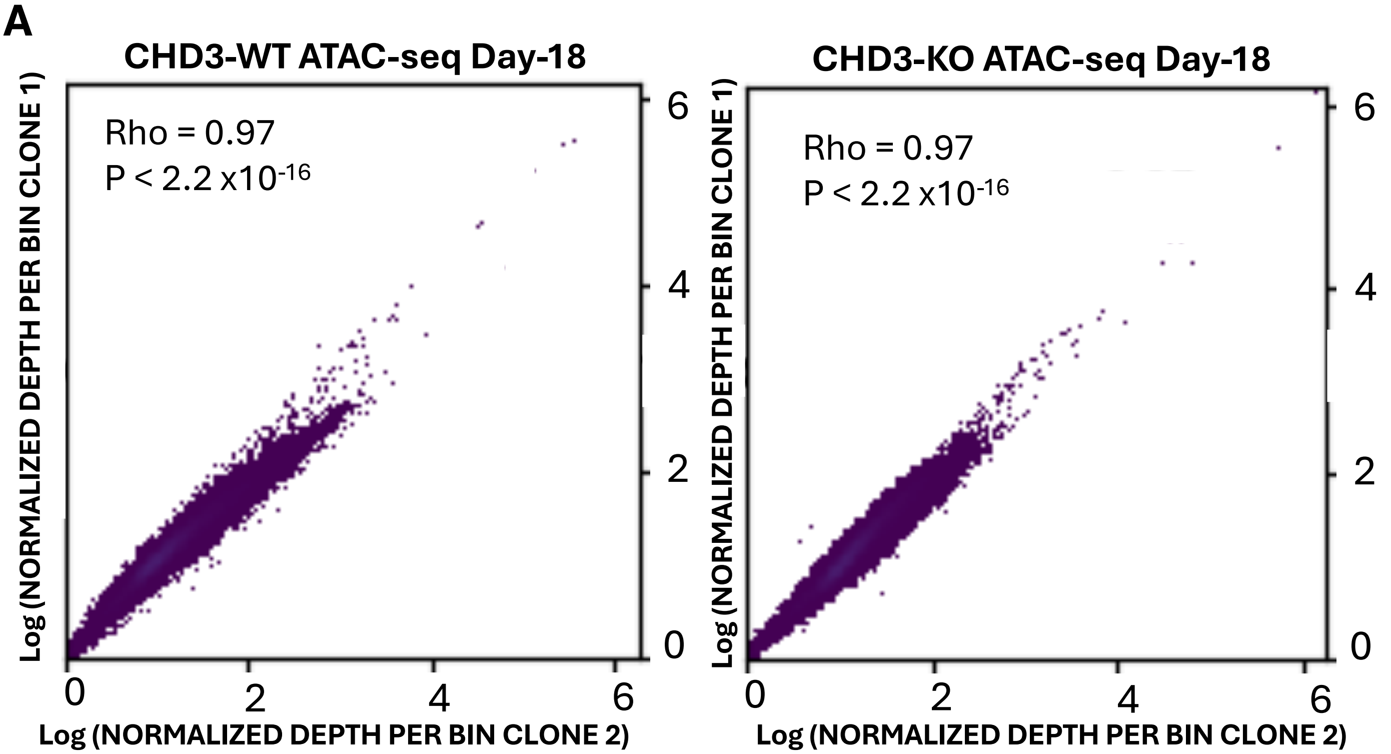

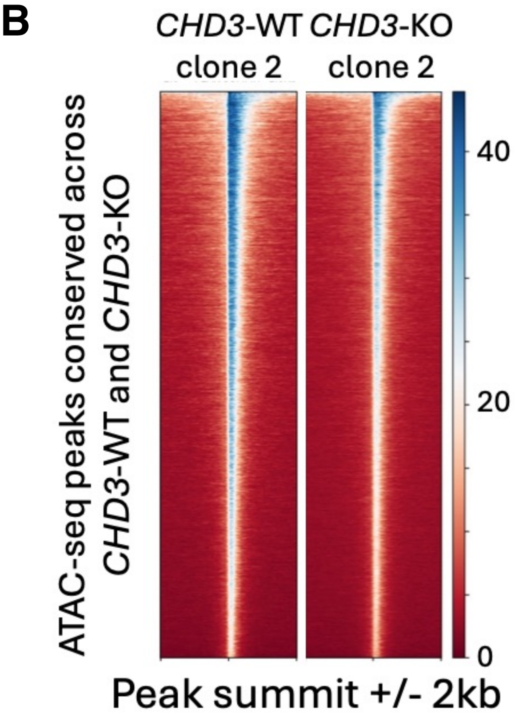

Appendix Figure S6. (A) Correlation plots showing spearman correlation across ATAC-seq biological replicates (i.e. *CHD3*-WT and *CHD3*-KO CRISPR-clones) at day 18 of iPSC-to-CNCC differentiation (bins = 1000bp). Exact p-values determined from spearman's correlation test are displayed. (B) Heatmap showing ATAC-seq peaks present in individual CHD3-WT and CHD3-KO replicates which are conserved in both *CHD3*-WT and *CHD3*-KO Day-18 CNCCs.

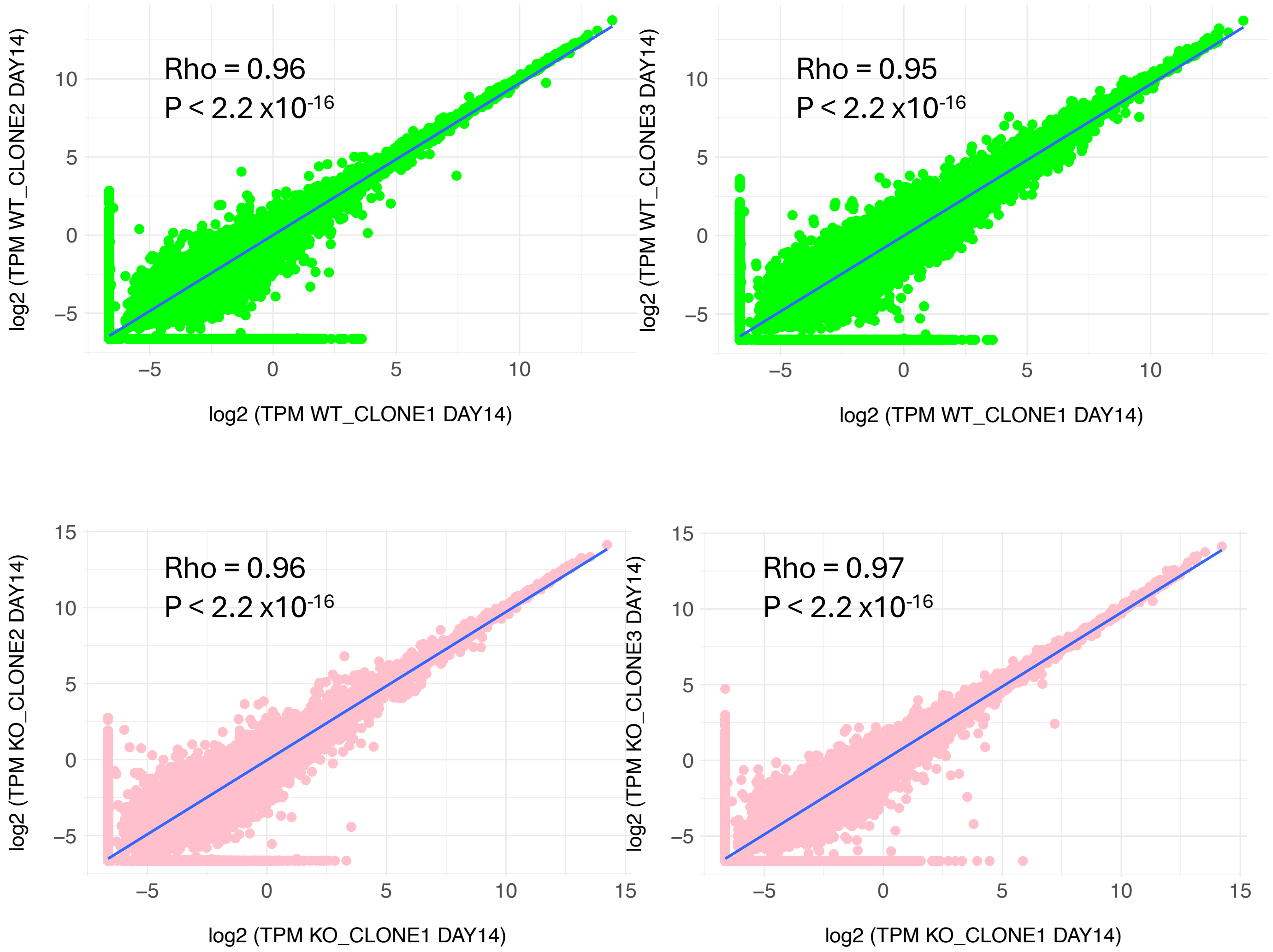

Appendix Figure S7. Correlation plots showing spearman correlation across RNA-seq biological replicates (i.e. *CHD3*-WT and *CHD3*-KO CRISPR-clones) at day 14 of iPSC-to-CNCC differentiation. Exact p-values determined from spearman's correlation test are displayed.

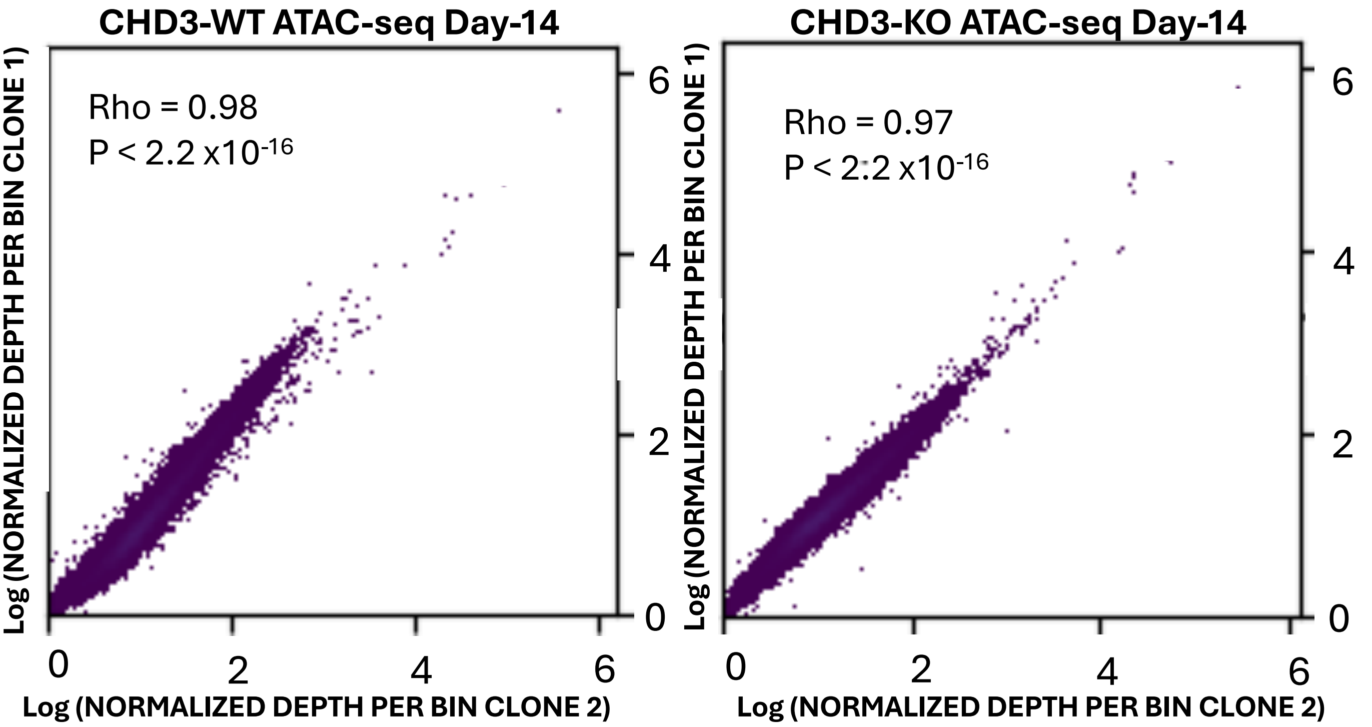

Appendix Figure S8. Correlation plots showing spearman correlation across ATAC-seq biological replicates (i.e. *CHD3*-WT and *CHD3*-KO CRISPR-clones) at day 14 of iPSC-to-CNCC differentiation (bins = 1000bp). Exact p-values determined from spearman's correlation test are displayed.

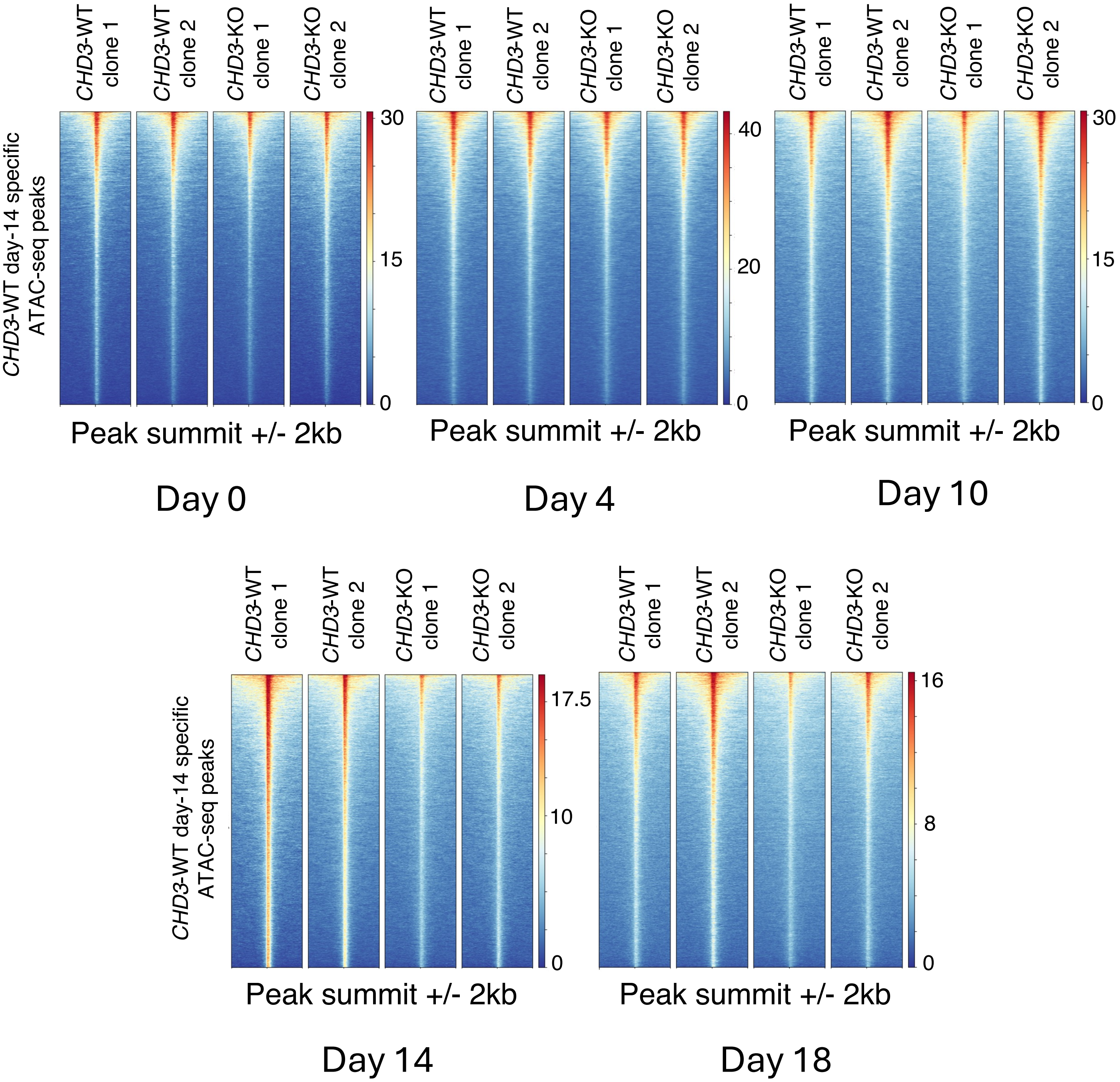

Appendix Figure S9. ATAC-seq time-course. Heatmaps showing ATAC-seq peaks present in individual CHD3-WT and CHD3-KO biological replicates at 5 different timepoints throughout iPSC to CNCC differentiation which are CHD3-WT specific in day-14 CNCCs.

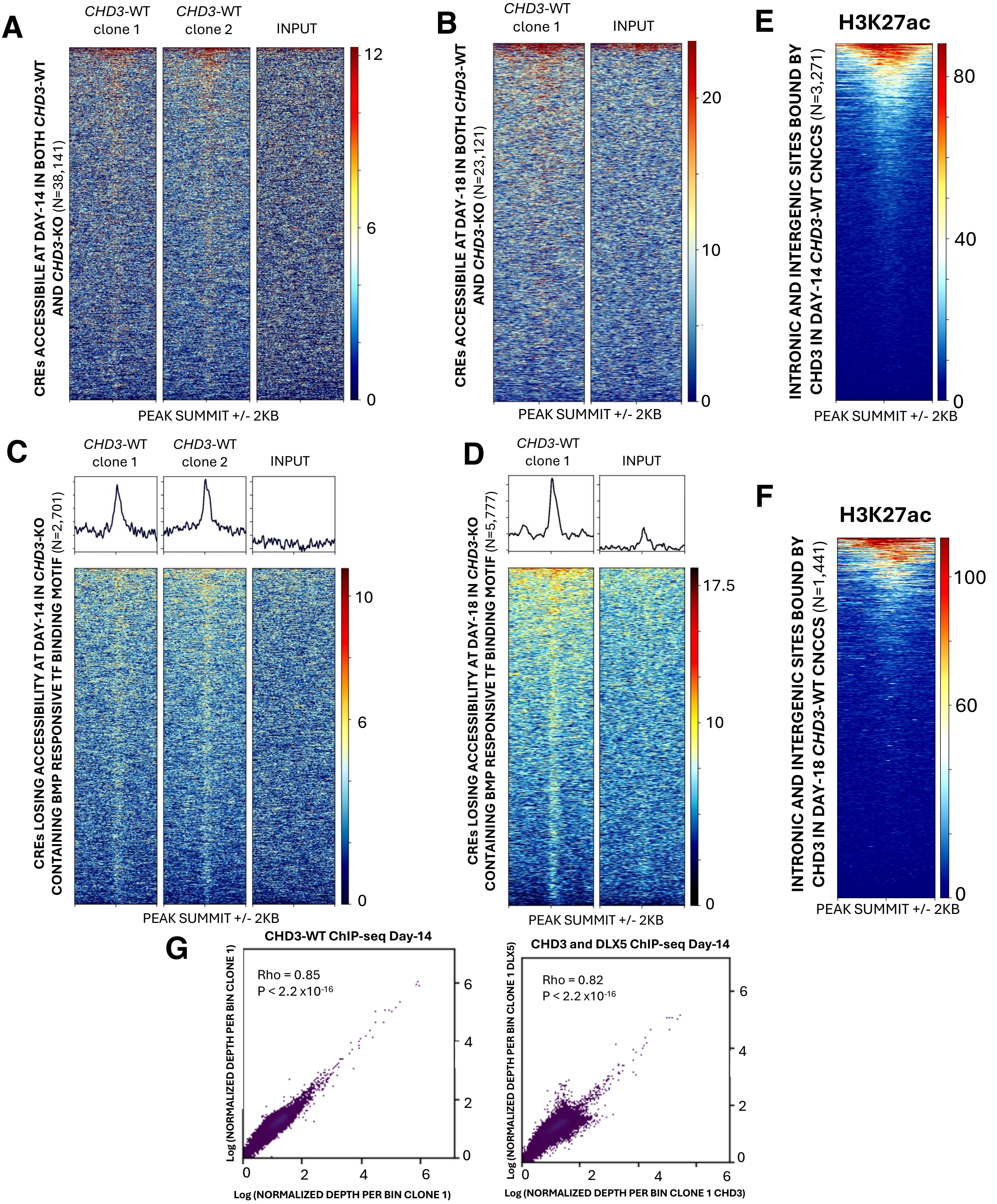

Appendix Figure S10. (A and B) Heatmaps of CHD3 binding at cis-regulatory elements (CREs) which are accessible in both *CHD3*-WT and *CHD3*-KO CNCCs at (A) day 14 or (B) day 18. (C and D) Heatmaps of CHD3 binding at cis-regulatory elements (CREs) which contain binding motifs for BMP responsive transcription factors (TF) and lose accessibility in (C) day 14 *CHD3*-KO CNCCs or (D) day 18 *CHD3*-KO CNCCs. (E and F) Heatmaps of H3K27ac, and indicator of active enhancers, at intronic and intergenic regions bound by CHD3 in *CHD3*-WT CNCCs at (E) day 14 and (F) day 18. (G) Correlation plots showing spearman correlation across CHD3 and DLX5 ChIP-seq biological replicates at day 14 of iPSC-to-CNCC differentiation (bins = 1000bp). Exact p-values determined from spearman's correlation test are displayed.

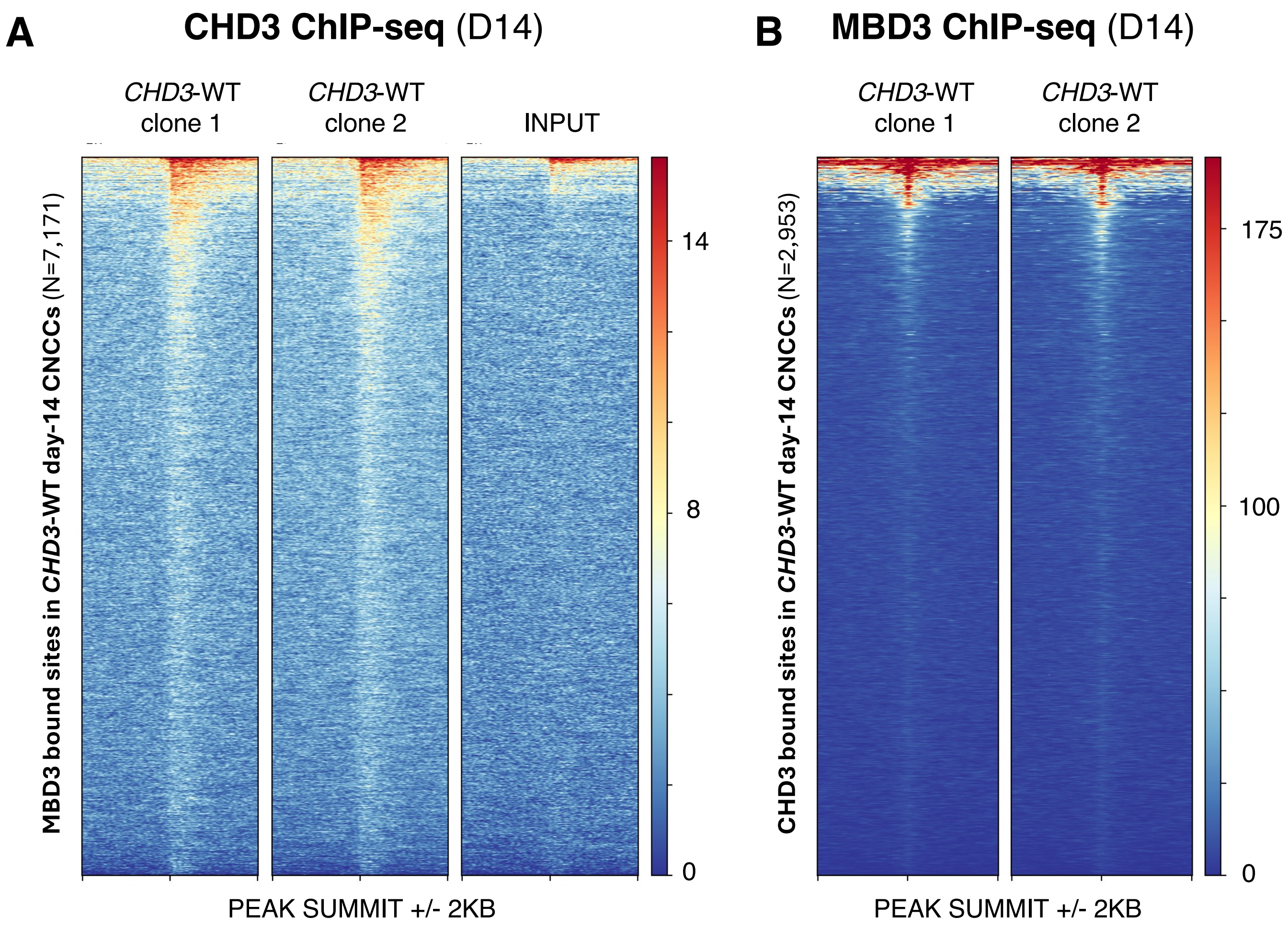

Appendix Figure S11. (A) Heatmap of CHD3 binding at sites bound by MBD3 in *CHD3*-WT CNCCs at day 14. (B) Heatmap of MBD3 binding at sites bound by CHD3 in *CHD3*-WT CNCCs at day 14.

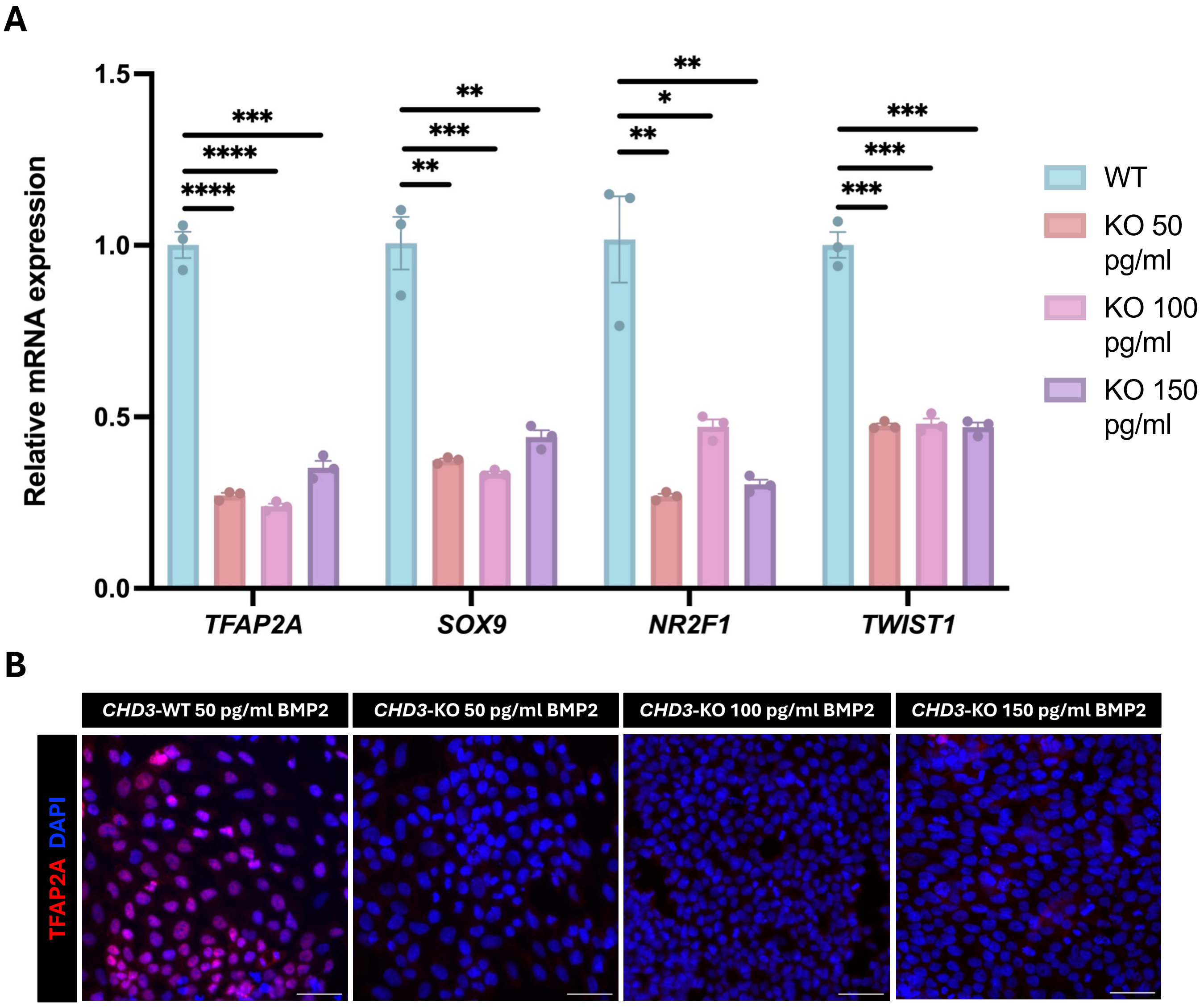

Appendix Figure S12. Increasing BMP2 dosage does not rescue CNCC marker expression in *CHD3*-KO D18 CNCCs. (A) RT-qPCR assessing the relative expression levels of CNCC markers (*TFAP2A*, *SOX9*, *NR2F1* and *TWIST*) between *CHD3*-WT (WT) and *CHD3*-KO (KO) D18 CNCCs provided with either the standard 50 pg/ml or an increased concentration (100 pg/ml or 150 pg/ml) of BMP2. *CHD3*-WT was given the standard 3 μM CHIRON and *CHD3*-KO was given 1 μM CHIRON (n=3 technical replicates for each condition). Differences between conditions were assessed using unpaired student's t-test. *=p<0.05, **=p<0.01, ***=p<0.001, ****=p<0.0001. Data are presented as mean ± SEM. Exact p-values: *TFAP2A*: *CHD3*-WT vs *CHD3*-KO 50pg/ml: p=0.00049, *CHD3*-WT vs *CHD3*-KO 100pg/ml: p=0.000041, *CHD3*-WT vs *CHD3*-KO 150pg/ml: p=0.000114. *SOX9*: *CHD3*-WT vs *CHD3*-KO 50pg/ml: p=0.001206, *CHD3*-WT vs *CHD3*-KO 100pg/ml: p=0.000965, *CHD3*-WT vs *CHD3*-KO 150pg/ml: p=0.002074. *NR2F1*: *CHD3*-WT vs *CHD3*-KO 50pg/ml: p=0.004053, *CHD3*-WT vs *CHD3*-KO 100pg/ml: p=0.012937, *CHD3*-WT vs *CHD3*-KO 150pg/ml: p=0.004891. *TWIST1*: *CHD3*-WT vs *CHD3*-KO 50pg/ml: p=0.000162, *CHD3*-WT vs *CHD3*-KO 100pg/ml: p=0.000217, *CHD3*-WT vs *CHD3*-KO 150pg/ml: p=0.000187. (B) Immunofluorescence for the CNCC marker TFAP2A in *CHD3*-WT and *CHD3*-KO D18 CNCCs provided with either the standard 50 pg/ml or an increased concentration (100 pg/ml or 150 pg/ml) of BMP2. *CHD3*-WT was given the standard 3 μM CHIRON and *CHD3*-KO was given 1 μM CHIRON. Scale bar: 50 μm.
