# Supplementary figures and images for: The NuRD component CHD3 promotes BMP signalling during cranial neural crest cell specification
Source: EMBO Rep. 2025 Aug 20;26(19):4723–41. doi: 10.1038/s44319-025-00555-w (PMC12508100; doi:10.1038/s44319-025-00555-w)

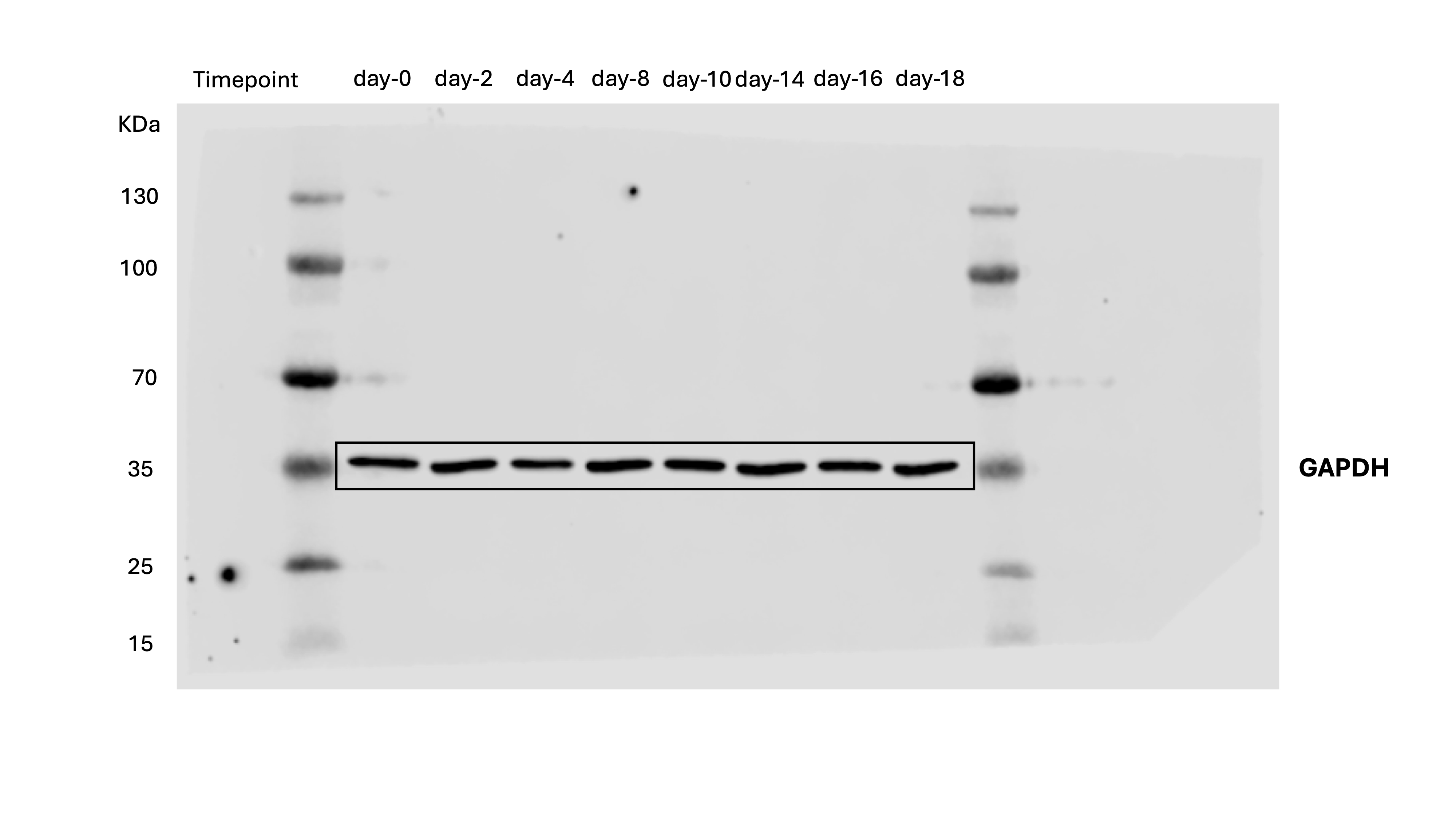

Supplement: Supplementary file 8 — Source data Fig. 1 [file 44319_2025_555_MOESM8_ESM.zip › EMBOR-2025-61878-T_SourceDataForFigure1/1F/GAPDH_western.jpg]

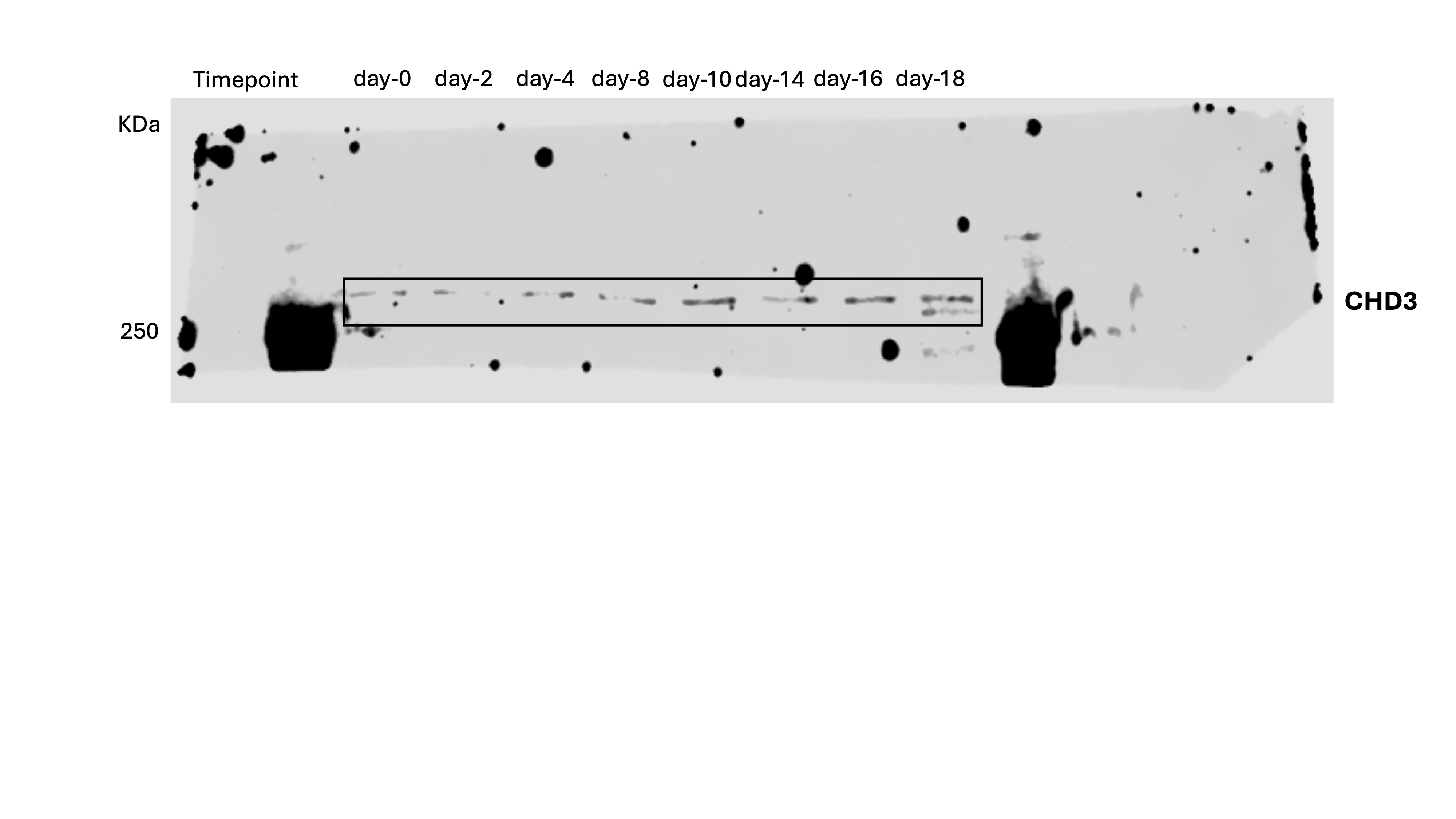

Supplement: Supplementary file 8 — Source data Fig. 1 [file 44319_2025_555_MOESM8_ESM.zip › EMBOR-2025-61878-T_SourceDataForFigure1/1F/CHD3_western.jpg]

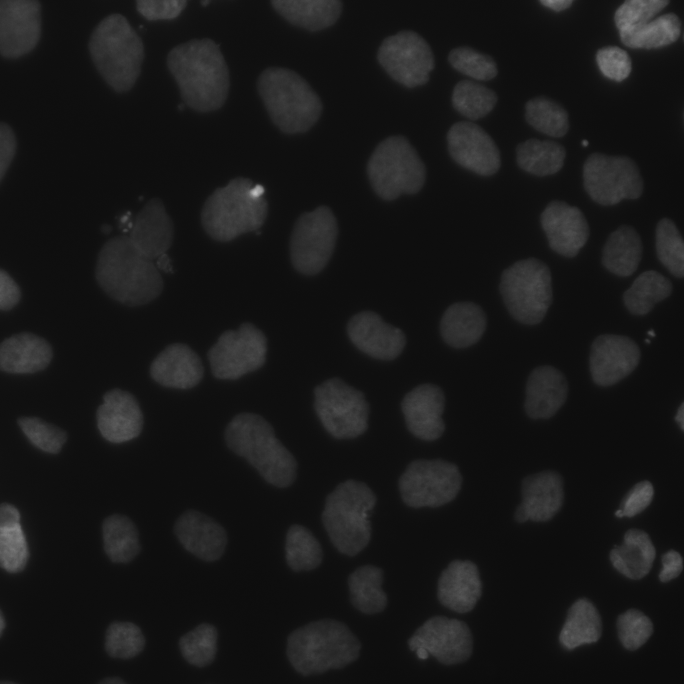

Supplement: Supplementary file 8 — Source data Fig. 1 [file 44319_2025_555_MOESM8_ESM.zip › EMBOR-2025-61878-T_SourceDataForFigure1/1E/CHD3_WT_d18_TFAP2A.tif]

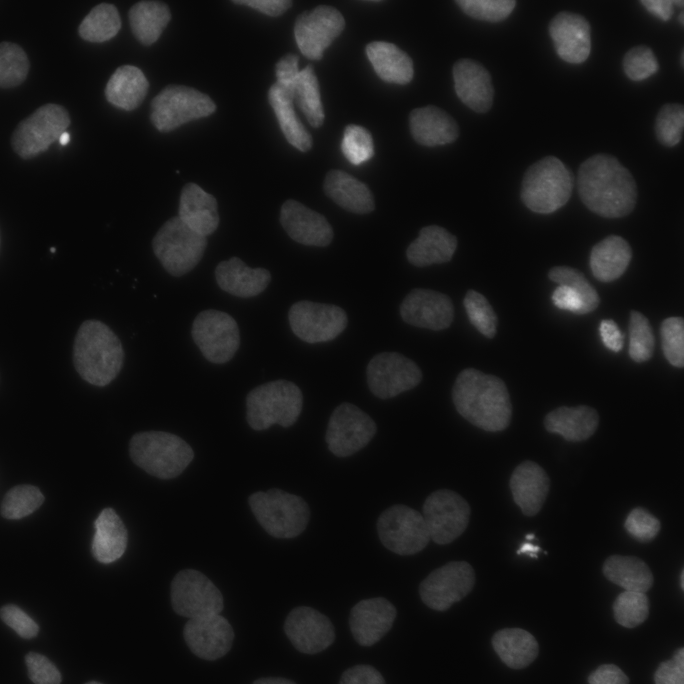

Supplement: Supplementary file 8 — Source data Fig. 1 [file 44319_2025_555_MOESM8_ESM.zip › EMBOR-2025-61878-T_SourceDataForFigure1/1E/CHD3_WT_d18_CHD3.tif]

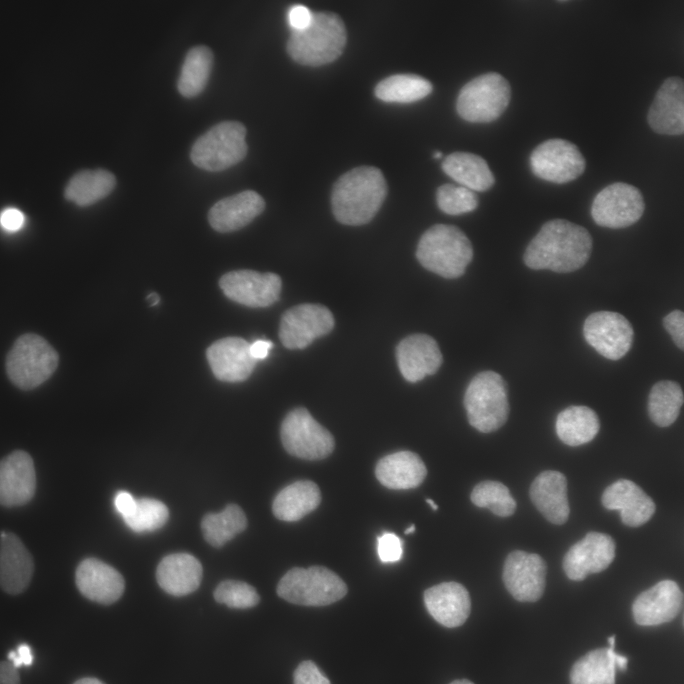

Supplement: Supplementary file 8 — Source data Fig. 1 [file 44319_2025_555_MOESM8_ESM.zip › EMBOR-2025-61878-T_SourceDataForFigure1/1E/CHD3_WT_d18_OCT4.tif]

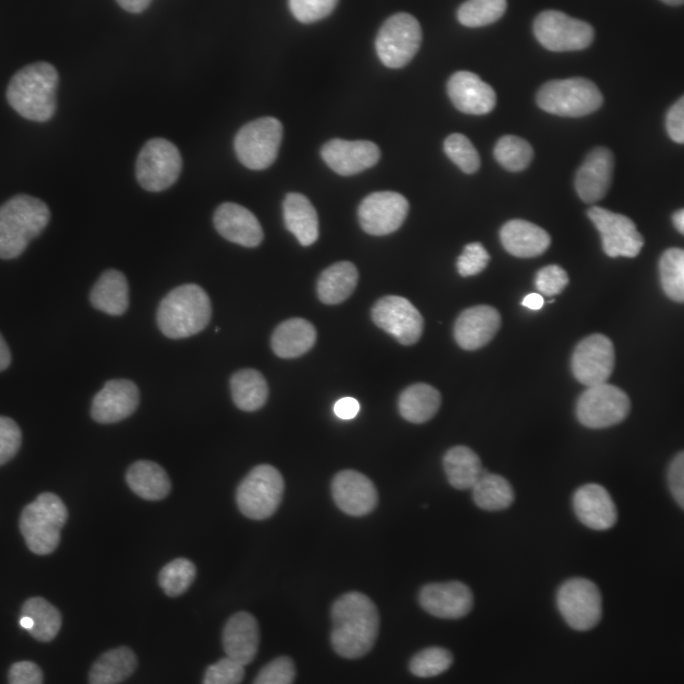

Supplement: Supplementary file 8 — Source data Fig. 1 [file 44319_2025_555_MOESM8_ESM.zip › EMBOR-2025-61878-T_SourceDataForFigure1/1E/CHD3_KO_d18_OCT4.tif]

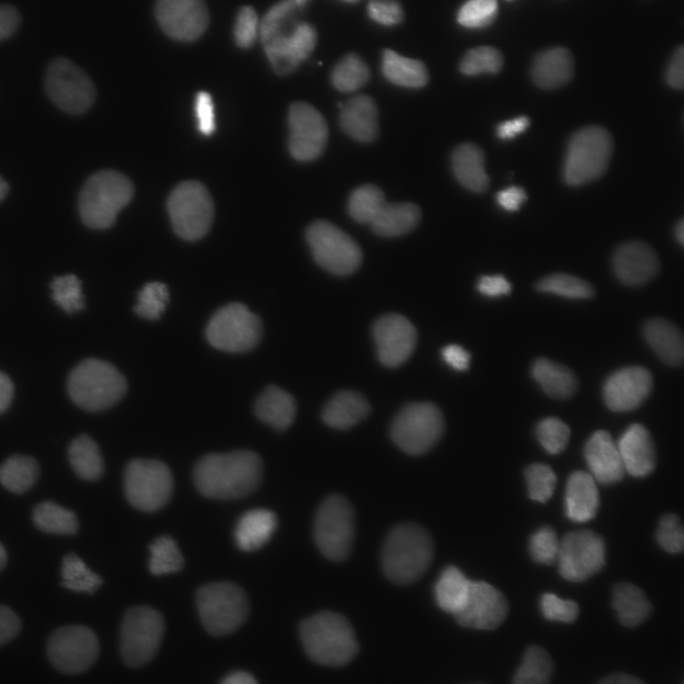

Supplement: Supplementary file 8 — Source data Fig. 1 [file 44319_2025_555_MOESM8_ESM.zip › EMBOR-2025-61878-T_SourceDataForFigure1/1E/CHD3_KO_d18_CHD3.tif]

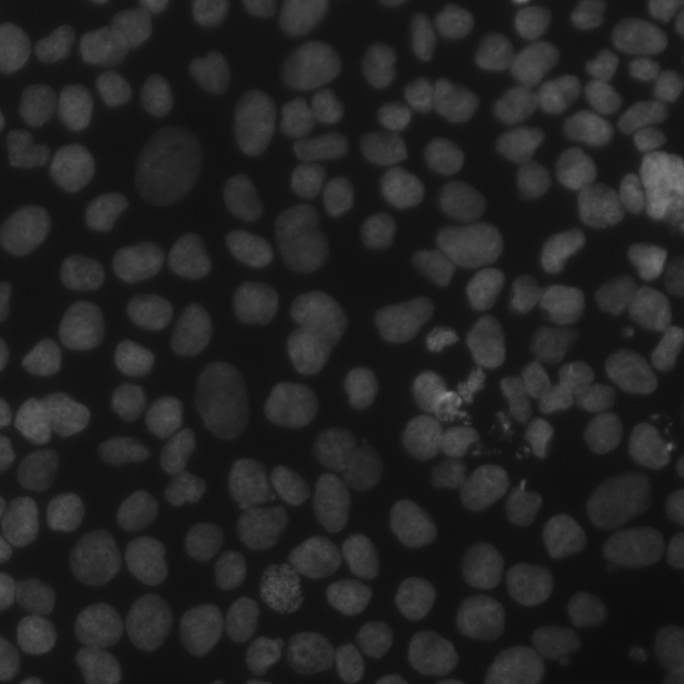

Supplement: Supplementary file 8 — Source data Fig. 1 [file 44319_2025_555_MOESM8_ESM.zip › EMBOR-2025-61878-T_SourceDataForFigure1/1E/CHD3_KO_d18_TFAP2A.tif]

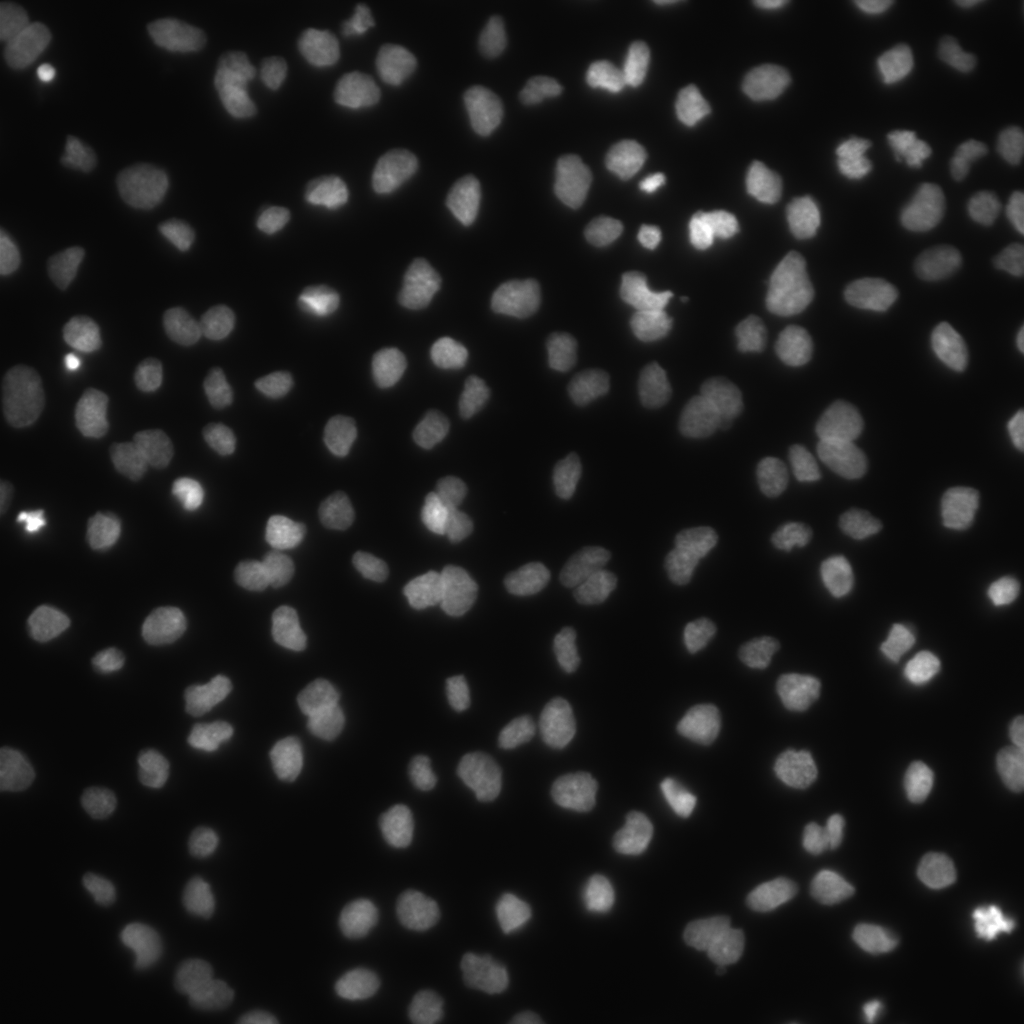

Supplement: Supplementary file 9 — Source data Fig. 2 [file 44319_2025_555_MOESM9_ESM.zip › EMBOR-2025-61878-T_SourceDataForFigure2/2D/CHD3_WT_d18_brachyury.tif]

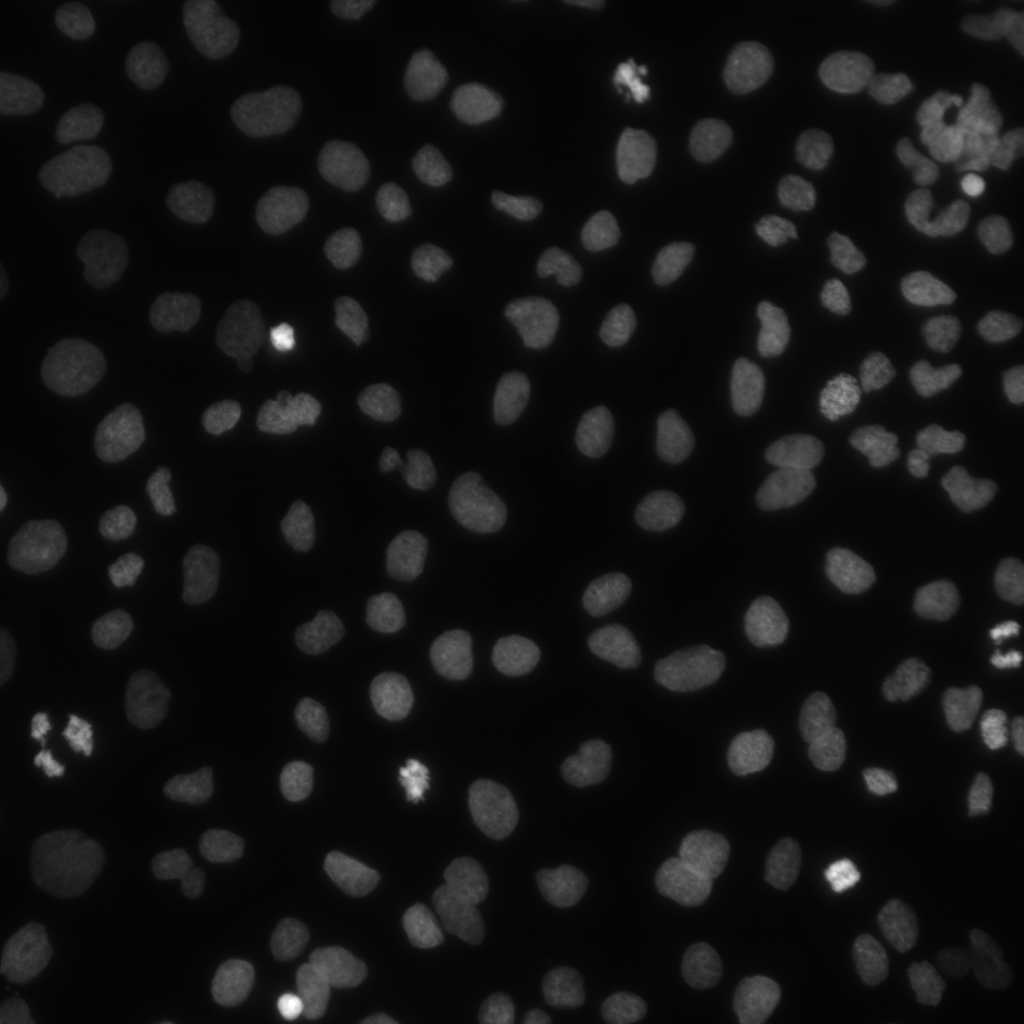

Supplement: Supplementary file 9 — Source data Fig. 2 [file 44319_2025_555_MOESM9_ESM.zip › EMBOR-2025-61878-T_SourceDataForFigure2/2D/CHD3_KO_d18_brachyury.tif]

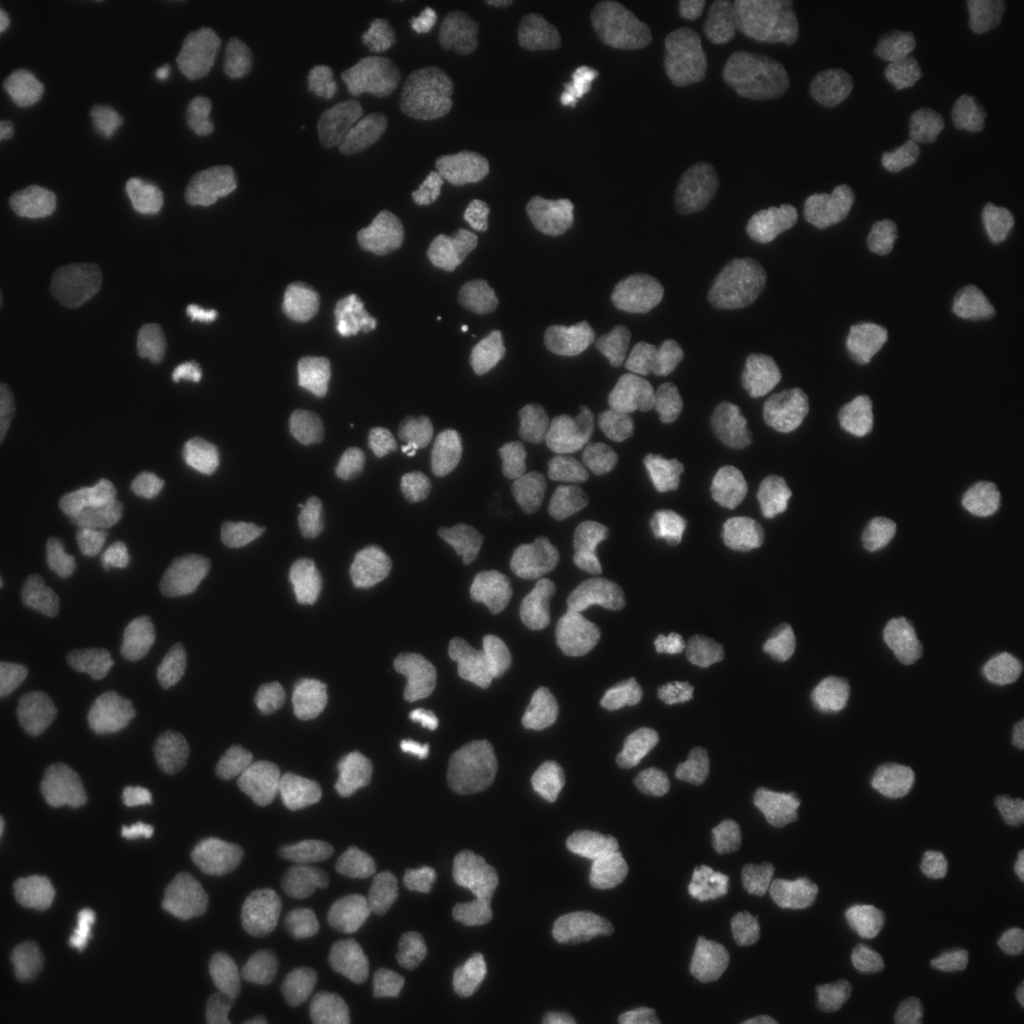

Supplement: Supplementary file 13 — Source data Fig. 6 [file 44319_2025_555_MOESM13_ESM.zip › EMBOR-2025-61878-T_SourceDataForFigure6/6B/CHD3_WT_d18_3um_chiron_TBX3.tif]

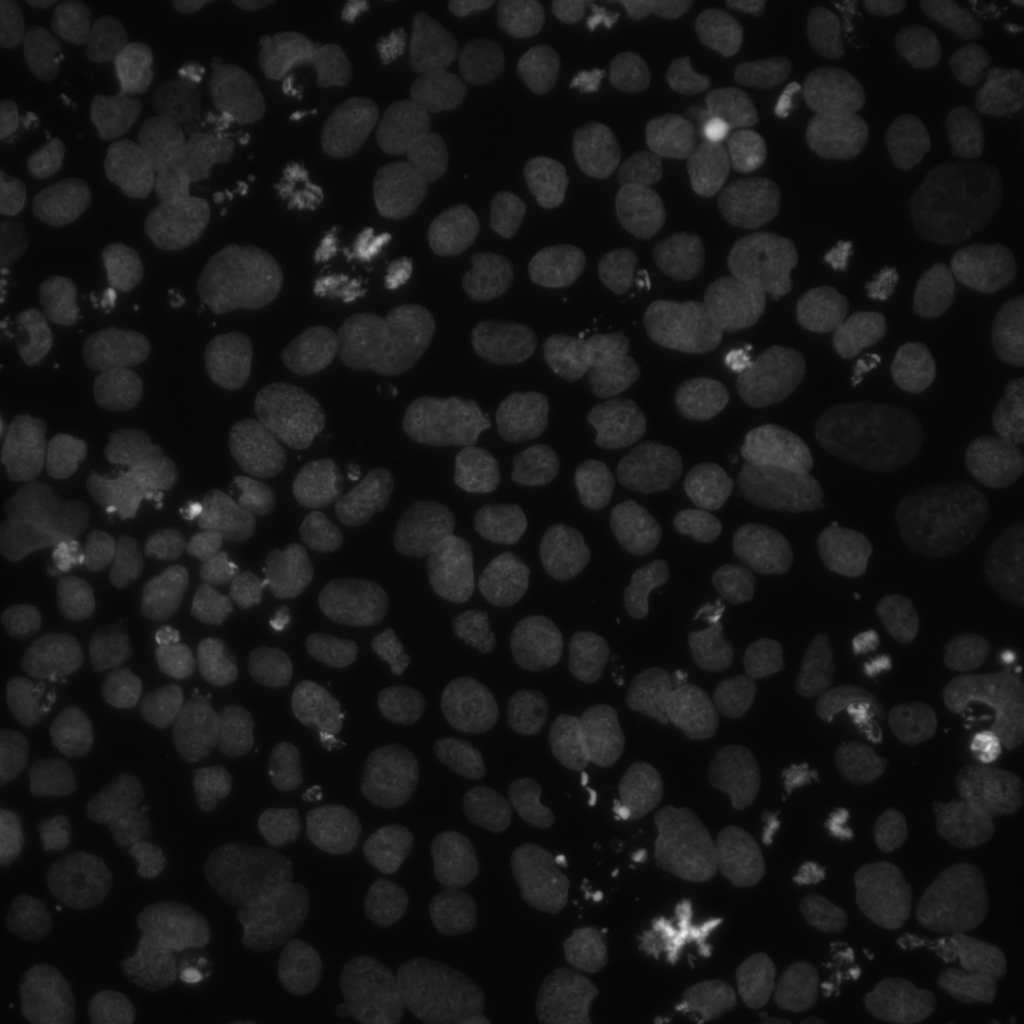

Supplement: Supplementary file 13 — Source data Fig. 6 [file 44319_2025_555_MOESM13_ESM.zip › EMBOR-2025-61878-T_SourceDataForFigure6/6B/CHD3_KO_d18_1um_chiron_TBX3.tif]

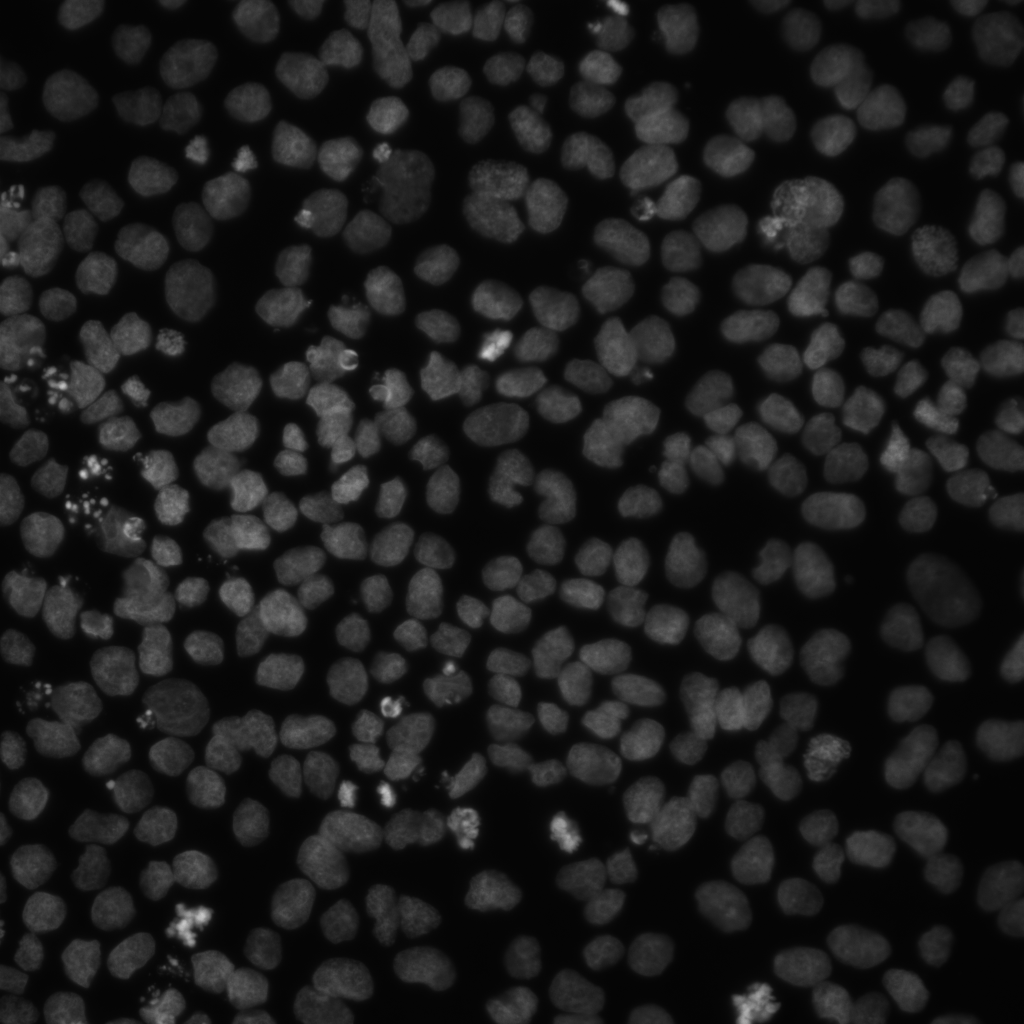

Supplement: Supplementary file 13 — Source data Fig. 6 [file 44319_2025_555_MOESM13_ESM.zip › EMBOR-2025-61878-T_SourceDataForFigure6/6B/CHD3_KO_d18_2um_chiron_TBX3.tif]

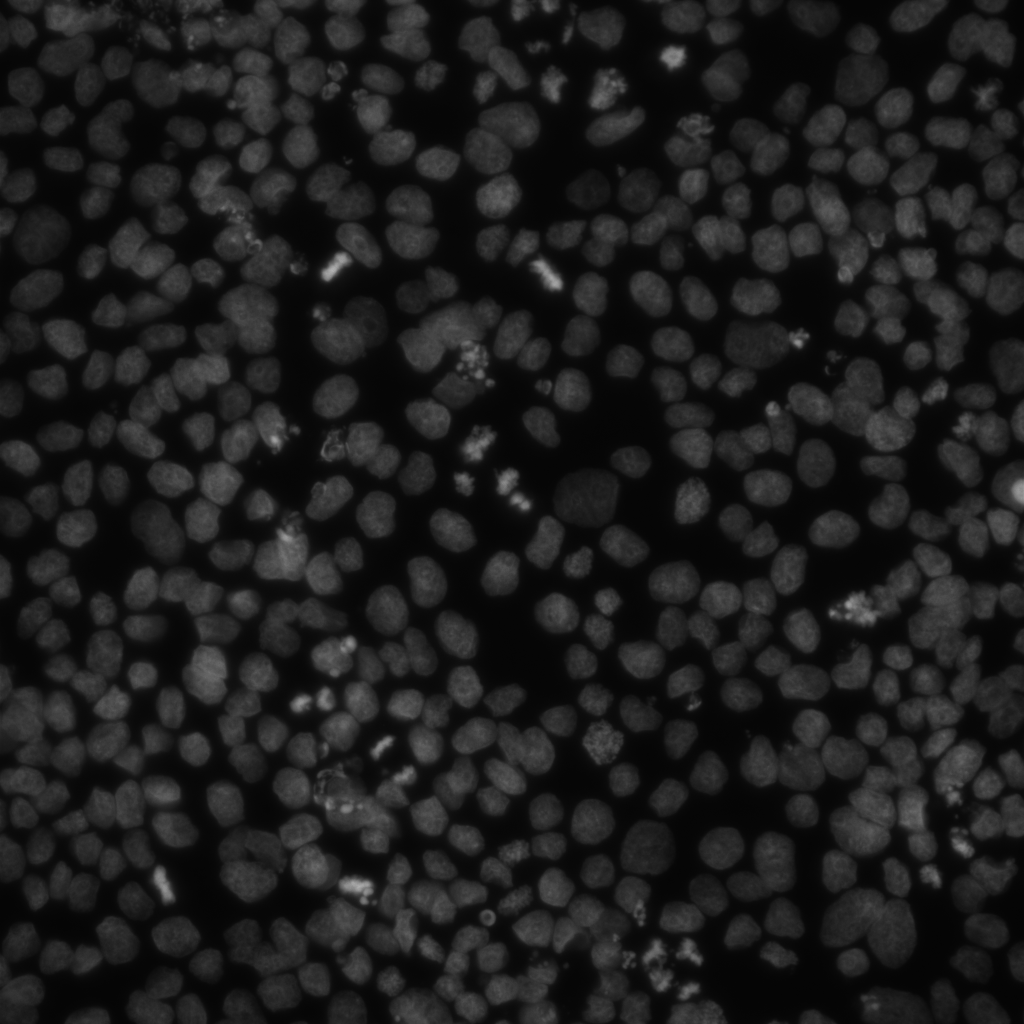

Supplement: Supplementary file 13 — Source data Fig. 6 [file 44319_2025_555_MOESM13_ESM.zip › EMBOR-2025-61878-T_SourceDataForFigure6/6B/CHD3_KO_d18_3um_chiron_TBX3.tif]
